# Furuncles

**Published:** 1893-11

**Authors:** 


### FURUNCLES.

Arnica. Small very painful boils. A small dose of Sulphur may be given to prevent a return.

The large ones, sometimes with second openings, usually require Hep., Lycop., or Nitric Acid; those with burning pains, Arsenic; the dark bluish-red, Lach.; the scarlet-red, Apis, Bellad., etc. All external treatment with medicinal salves and plasters is not only perfectly useless, but tend to obscure the external indications for the selection of the remedy as illustrated by the above.

